# Evaluation of hypermobility syndrome associated with vesico-ureteric reflux in children

**DOI:** 10.1186/1546-0096-9-S1-P240

**Published:** 2011-09-14

**Authors:** Reza Shiari, Reza Amir Askari, Masumeh Mohkam

**Affiliations:** 1 Shahid Beheshti University of Medical Sciences, Mofid Children Hospital, Tehran-Iran; 2 Pediatric Infectious Research Center (PIRC), Tehran-Iran

## Background

Joint hypermobility Syndrome is an inherited disease of connective tissue with symptoms of musculoskeletal involvement. On the other hands, Idiopathic reflux occurs in approximately 1% of the children and cause kidney scar and hypertension.

## Aims

Considering the etiology of hypermobility syndrome that is involvement of connective tissue as a result of a disruption in the synthesis of collagen, there is a possibility of other organs involvement such as urinary system which the structure of connective tissue plays an important role. The aim of this study was to determine the association of hypermobility syndrome with vesico*-*ureteric reflux in children.

## Materials and methods

This research was performed on 70 children between 3-16 years old with vesico-ureteric reflux and 70 normal children as a case control study.

## Result

There was an association between urinary tract infection and hypermobility syndrome (P=0.001). Urinary reflux had a significant relation with myalgia (P≤.0.026). There was an association between myalgia and arthralgia, with hypermobility syndrome (P≤.0.001) and (P=0.03) respectively. We found a significant association between vesico-ureteric reflux and hypermobility syndrome (P≤0.001).

## Conclusion

Considering the significant relation of vesico-ureteric reflux and hypermobility syndrome, it’s been recommended that children with hypermobility syndrome are better evaluated for urinary tract infection.

